# Evaluation of light output from light-curing units used in a public oral health service in Brazil

**DOI:** 10.1590/0103-644020267001

**Published:** 2026-08-24

**Authors:** Laryssa Silva da Cunha, Airin Karelys Avendaño Rondon, Maryuri del Carmen Macias Bautista, Ianca Daniele Oliveira de Jesus, Guilherme Mendonça Benoni, Gisele Rodrigues da Silva, Richard Bengt Price, Carlos José Soares

**Affiliations:** 1 Department of Operative Dentistry and Dental Materials, School of Dentistry, Federal University of Uberlândia, Uberlândia, Minas Gerais, Brazil; 2 Department Dental Clinical Sciences and School of Biomedical Engineering, Dalhousie University, Nova Scotia, Halifax, Canada

**Keywords:** Light, oral health, polymerization, resin composites

## Abstract

This observational field study aimed to evaluate the radiant power (mW), emission spectra (mW/cm²/nm), irradiance (mW/cm²), and the light tip integrity of 40 light-curing units (LCUs) used in 16 public oral health service units in the city of Uberlândia, Minas Gerais, Brazil. The radiant power and emission spectrum were measured using a spectrometer attached to an integrating sphere. Irradiance profiles were obtained using a Beam Profiler. The tips were photographed and classified in 4 categories: A. undamaged, B. contaminated by resin residue, C. damaged, and D. contaminated and damaged. Data were analyzed by one-way ANOVA (α = 0.05), and the emission spectra and beam profiles were analyzed descriptively. The LCUs tested were single-peak light-emitting diode units, and 85% of the LCUs had damaged light tips. The radiant power values were significantly different between the LCUs (P < 0.001) and ranged between 94.2 and 588.2 mW. The irradiance ranged from 255.8 to 1597.8 mW/cm². The LCU tip diameter ranged from 6.1 to 8.3 mm. Two-dimensional beam profiles show that the light distribution was not homogeneous across the tips, and an irradiance peak was often observed at the center of the LCUs with smaller tips. Only 15% of the LCU were undamaged, reflecting substantial variability in radiant power and irradiance delivered. Although no statistically significant differences were observed among tip condition categories, these conditions showed a trend toward reduced and inhomogeneous light output.

## Resumo

Este estudo observacional de campo teve como objetivo avaliar a potência radiante (mW), espectro de emissão (mW/cm²/nm), irradiância (mW/cm²) e integridade da ponta de 40 aparelhos de fotopolimerização (FP) utilizadas em 16 unidades públicas de saúde bucal na cidade de Uberlândia, Brasil. A potência radiante e o espectro de emissão foram medidos utilizando espectrômetro acoplado a esfera integradora. Os perfis de irradiância foram obtidos utilizando perfilador de feixe de luz. As pontas das FPs foram fotografadas e classificadas em 4 categorias: A. intactas, B. contaminadas por resíduos de resina, C. danificadas e D. contaminadas e danificadas. Os dados foram analisados por ANOVA de um fator (α = 0,05), e os espectros de emissão e os perfis de feixe foram analisados descritivamente. Os FPs testados eram unidades de diodo emissor de luz de pico único de luz (azul), e 85% das pontas de luz dos FPs foram classificadas como contaminadas, danificadas ou ambas. Os valores de potência radiante foram significativamente diferentes entre os FPs (P < 0,001) e variaram entre 94,2 e 588,2 mW. A irradiância variou de 255,8 a 1597,8 mW/cm². O diâmetro da ponta variou de 6,1 a 8,3 mm. Os perfis do feixe de luz mostraram distribuição não homogênea entre as pontas. Frequentemente observou-se pico de irradiância no centro da ponteira. Apenas 15% das LCUs estavam intactas, apresentando variabilidade significativa na potência radiante e na irradiância. Embora não tenham sido observadas diferenças estatisticamente significativas entre as categorias de condição das pontas, essas condições estavam associadas à emissão de luz reduzida e não homogênea.

**Figure:**
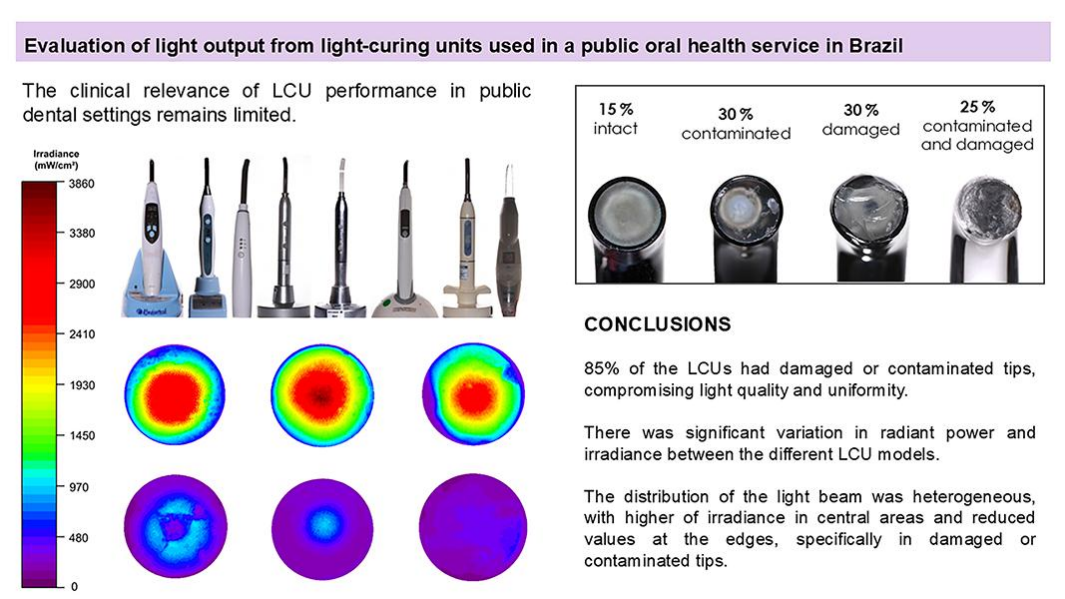

## Introduction

It is estimated that over 800 million resin-based composite (RBC) restorations are placed worldwide each year^1^. Light-curing is a critical step in restorative dentistry, and a good light-curing unit (LCU) is essential for achieving the optimal mechanical properties and longevity of RBC restorations, resin cements, and sealants^2^. The light-curing effectiveness depends on factors related to the light source, including the type of LCU, irradiance, energy delivered, emission spectrum, beam profile, and the light tip diameter^3^. Clinical factors, such as the operator's technique and the quality of LCU maintenance, can also affect the light-curing of the resin composite^4^ ^,^ ^5^.

Irradiance (mW/cm²) is a key parameter for adequate monomer conversion^6^ ^,^ ^7^. Manufacturers recommend minimum exposure times (e.g., 10 seconds) and minimum irradiance levels ^3^ ^,^ ^6^. However, since irradiance is calculated as power per unit area, it must be recognized that this represents an average over the surface. It assumes that a uniform light beam will be delivered from the LCU tip to ensure adequate polymerization across the entire surface of the restoration ^8^. Most LCUs exhibit non-uniform beam profiles, with a higher irradiance at the center and a lower irradiance at the tip edges ^4^ ^,^ ^9^. This non-uniform light distribution can negatively affect polymerization and increase the risk of adhesive failures ^10^. Inadequate maintenance, physical damage, and contamination of the tip can reduce irradiance and compromise light-curing effectiveness ^11^ ^,^ ^12^ ^,^ ^13^. 

The clinical relevance of LCU performance in public dental settings remains limited ^13^ ^,^ ^14^. The Brazilian Unified Health System (Sistema Único de Saúde - SUS), one of the world's largest public health systems, provides universal access to free healthcare services ^15^ ^,^ ^16^. The use of amalgam in the public service is declining ^16^. In Uberlândia, Minas Gerais, Brazil, its use was eliminated 2 years ago. RBCs have been gaining popularity due to their aesthetic appeal and adhesive properties, replacing amalgam ^1^. Consequently, the use of LCUs has also increased substantially. 

The quality of LCUs influences the light-curing process and the longevity of restorations. Therefore, this study measured radiant power (mW), emission spectra (mW/cm²/nm), irradiance (mW/cm²), and tip integrity for 40 LCUs used in 16 public oral health service units in the city of Uberlândia, Brazil. The null hypotheses were that: ^1^ the LCUs used in the public health service would deliver similar radiant powers and irradiance values, and ^2^ the LCUs would show no signs of tip damage or contamination.

## Methodology

The institutional ethics committee (80002924.2.0000.5152) approved the research protocol. This observational field study was conducted in 16 public oral health service units in 5 regions of the city of Uberlândia, Minas Gerais (MG), Brazil. The public oral health units were selected using a convenience sampling approach, based on the availability of LCUs in clinical use at the time of evaluation. To ensure broader representation of the public oral health service, units from different geographical regions of the city were included. Forty LCUs were evaluated. Information on the duration of use, the number of curing cycles, and the maintenance history of the LCUs was not available. The inclusion criteria were public oral health units with LCUs in clinical use at the time of evaluation. The exclusion criteria were LCUs that were unavailable, not in use, or could not be assessed under standardized conditions. Monthly averages of RBC restorations provided by the health unit were collected to estimate clinical demand and LCU's use.

Light tip damage, diameter, and area calculation

The internal and external diameters (mm) of the light tips were measured with a digital caliper (Mitutoyo, Tokyo, Japan), and the light tip area (mm²) was calculated. The LCUs were photographed to record damage and contamination by residues on the light tip. Each tip was classified by a single trained operator using predefined objective visual criteria, under standardized conditions, based on photographic records and direct visual inspection. The following categories were used: intact, contaminated by residues, damaged, or both contaminated and damaged ^17^.

Total radiant power and emission spectrum

The power (mW) and spectral radiant power (mW/nm) were recorded using a fiber optic spectrometer (USB 4000; Ocean Insight, Orlando, USA) connected to a six-inch integrating sphere (LabSphere; North Sutton, USA). All LCUs were fully charged. Ten measurements for each LCU were recorded with the LCU tip at 0 mm through a 12 mm diameter entrance to the integrating sphere to ensure complete capture of all light emitted from the LCU tip.

Beam profile

The light distribution across the light tip was measured using a laser beam profiler (SP620U; Ophir-Spiricon, Logan, USA). The LCUs were mounted 0 mm from a 60 ° holographic diffusing screen (54-505, Edmund Optics, Barrington, USA). Two blue filters (HOYA B410 bandpass filters; #34-434 Edmund Optics) and reflective neutral density filters (Edmund Optics) were used. The images were collected using the beam analyzer software (BeamGage Professional version 6.21.1; Ophir-Spiricon, Logan, USA). The “Optical Scaling” tool calibrated the beam profile dimensions, and the data were exported to OriginPro 2025 version 10.2. (OriginLab; Northampton, USA).

Statistical analysis

The radiant power (mW), irradiance (mW/cm²), and tip diameter (mm) were analyzed for normal distribution and homoscedasticity using the Shapiro-Wilk and Levene’s tests. The power data were analyzed using one-way ANOVA. Tip damage and residues were analyzed descriptively by calculating the absolute and relative frequencies for each category observed at the tips of the LCUs. Additionally, irradiance values were compared across light tip condition categories (intact, contaminated, damaged, and both contaminated and damaged) using a one-way ANOVA, followed by Tukey's post hoc test. All tests used a significance level of α = 0.05 and were performed with Sigma Plot 13.1 (Systat Software Inc., San Jose, USA). The emission spectra (nm/mW/cm²), beam profiles, and the mean RBC restoration rates in the public health units were analyzed descriptively. 

## Results

The information on the public oral health service units and the mean monthly number of RBC restorations delivered from January to June 2025 is reported in Table 1. The overall mean among the 16 units was 132 RBC restorations a month.

**Table 1:** Number of LCUs in each Basic Health Unit and the average number of restorations placed each month.

Basic health unit		City region	N. of LCUs	Average number of RBC restorations
UAI Tibery		East	3	188
UBSF Dom Almir		East	4	123
UAI São Jorge		South	2	199
UBS Patrimônio		South	2	24
UBSF Joana Darc		East	2	177
UBSF Morumbi 1 e 2		East	3	86
UBSF Aclimação		East	2	85
UBS Brasil		Center	2	148
UBSF Monte Hebron		West	3	92
UBSF Dona Zulmira		West	2	136
UBSF Jardim Brasília 2		North	3	111
UAI Martins		Center	4	177
UAI Roosevelt		North	2	170
UAI Luizote		West	3	240
UBSF Bom Jesus		Center	2	47
UBSF Santa Rosa		North	1	107
Total	16	5	40	132

The specifications and prices of the LCUs in Brazil are reported in Table 2.

**Table 2:** The specifications of LCUs measured in this study.

N. of LCUs	Name/ cost (US$)	Basic health units	Serial number	LCU wavelength emission	Battery/mains	Tip/light conductor	Manufacturer
8	LED 6 121.5	East	LD61904733R	Single-peak	Battery	Optical fiber/ black	Kondentech, São Carlos, SP, Brazil
		Center	LD61904732R				
		East	LD61904729R				
		South	LD61904739R				
		South	LD61904737R				
		South	LD621011034R				
		South	LD61904730R				
		West	LD61904726R				
17	Prime Led 3 101.8	West	321383	Single-peak	Battery	Optical fiber/ black	Dentemed, Belo Horizonte, MG, Brazil
		West	321482				
		East	319095				
		East	319105				
		East	319100				
		East	319099				
		East	278310				
		East	319495				
		East	319496				
		West	319109				
		West	319104				
		North	321498				
		North	321499				
		Center	329586				
		Center	329585				
		North	319094				
		North	319089				
2	Optilight Max 235.4	East	307950	Single-peak	Battery	Optical fiber/ black	Gnatus, Ribeirão Preto, SP, Brazil
		West	312898				
b2	Optilight Max 305.4	East	229743	Single-peak	Battery	Optical fiber/ black	Dabi Atlante, Ribeirão Preto, SP, Brazil
		East	229742				
1	LuxCler LED 130.0	Center	231183	single-peak	Battery	Optical fiber/ black	Dentscler, Ribeirão Preto, SP, Brazil
3	DB686 142.3	Center	201707080	single-peak	Battery	Optical fiber/ Black	Dabi Atlante, Ribeirão Preto, SP, Brazil
		Center	201303030				
		East	201303026				
6	Emitter A. fit 127.0	Center	L1903418S	single-peak	Battery	Optical fiber/ black	Schuster, Santa Maria, RS, Brazil
		North	L2232862S				
		West	L20B3112S				
		West	L2232861S				
		East	L1003435S				
		North	L20B3168S				
1	LD Max 235.4	Center	None	single-peak	Mains	Plastic/ translucent	Gnatus, Ribeirão Preto, SP, Brazil

### Light Tip Area

**Figure 1:**
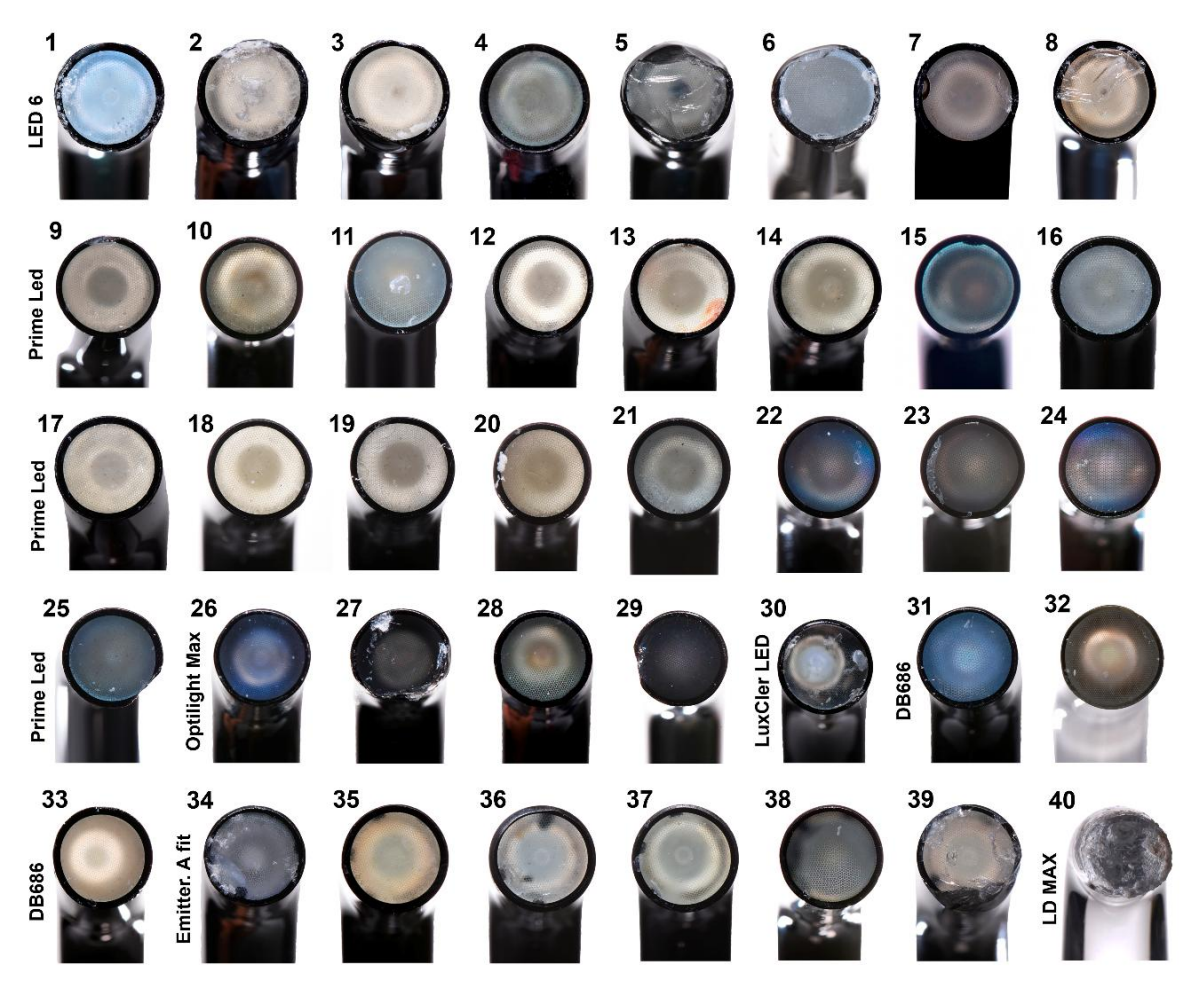
Images of the different light tips: 1 to 8: LED 6; 9 to 25: Prime Led 3; 26 to 29: Optilight Max; 30: LuxCler LED; 31 to 33: DB686; 34 to 39: Emitter A. fit and 40: LD Max. The light tips were classified into the following categories: intact, contaminated with some residue, damaged, and both contaminated and damaged. Note the contamination and damage on the LCU tips.

The external tip diameter ranged from 7.7 to 8.3 mm, and the internal tip diameter ranged between 6.1 and 8.3 mm (Table 3). The condition of the light tips indicated that several were damaged, and resin residue was present on many of them (Figure 1). Only 6 LCUs (15%) had undamaged light tips, 12 LCUs (30%) were contaminated by some residue, 12 (30%) were damaged, and 10 (25%) were both contaminated and damaged. 

**Table 3:** Mean and standard deviation (SD) of the internal and external tip diameters (mm), mean (± SD) for radiant power (mW), irradiance (mW/cm²), and wavelength peaks (nm) of the 40 LCUs. Values represent the mean ± standard deviation of 10 measurements per LCU.

Code	Name	Mean ± SD external tip diameter (mm)	Mean ± SD internal tip diameter (mm)	LCU wavelength peaks (nm)	Mean ± SD radiant power (mW)	Mean ± SD irradiance (mW/cm^2^)
1	LED 6	8.1 ± 0.1	6.8 ± 0.1	457	588.2 ± 12.8	1597.8 ± 34.9
2	LED 6	8.0 ± 0.1	6.9 ± 0.1	458	181.4 ± 1.4	480.8 ± 3.7
3	LED 6	8.3 ± 0.1	7.1 ± 0.1	457	492.5 ± 8.3	1232.6 ± 20.7
4	LED 6	8.1 ± 0.1	6.9 ± 0.1	455	434.5 ± 10.9	1156.6 ± 29.0
5	LED 6	8.1 ± 0.1	7.0 ± 0.1	450	206.2 ± 2.8	542.3 ± 7.5
6	LED 6	7.8 ± 0.2	7.1 ± 0.1	455	313.9 ± 0.4	793.2 ± 1.0
7	LED 6	8.1 ± 0.1	6.9 ± 0.1	455	432.3 ± 6.8	1120.6 ± 18.1
8	LED 6	7.8 ± 0.1	7.1 ± 0.1	451	225.6 ± 4.7	577.8 ± 12.0
9	Prime Led 3	7.8 ± 0.1	6.8 ± 0.1	452	361.9 ± 3.4	992.8 ± 9.2
10	Prime Led 3	7.9 ± 0.1	6.9 ± 0.1	446	251.8 ± 1.8	665.9 ± 4.8
11	Prime Led 3	8.1 ± 0.1	7.0 ± 0.1	447	395.1 ± 3.1	1032.4 ± 8.1
12	Prime Led 3	8.1 ± 0.1	7.1 ± 0.1	447	361.6 ± 3.2	915.4 ± 8.2
13	Prime Led 3	8.0 ± 0.1	6.8 ± 0.1	449	309.1 ± 3.2	845.4 ± 8.9
14	Prime Led 3	8.1 ± 0.1	6.8 ± 0.1	446	327.7 ± 2.5	893.3 ± 6.9
15	Prime Led 3	8.1 ± 0.1	6.9 ± 0.1	451	265.2 ± 3.9	714.9 ± 10.6
16	Prime Led 3	8.0 ± 0.1	6.6 ± 0.1	447	306.1 ± 2.6	906.8 ± 7.6
17	Prime Led 3	8.0 ± 0.1	6.7 ± 0.1	447	330.1 ± 2.4	932.2 ± 6.9
18	Prime Led 3	7.7 ± 0.3	6.7 ± 0.1	448	340.1 ± 3.3	965.1 ± 9.3
19	Prime Led 3	8.1 ± 0.1	6.7 ± 0.1	447	378.4 ± 3.2	1057.8 ± 9.0
20	Prime Led 3	8.3 ± 0.1	7.0 ± 0.1	447	328.3 ± 3.8	848.9 ± 9.9
21	Prime Led 3	8.1 ± 0.1	6.8 ± 0.1	451	370.4 ± 2.3	1013.3 ± 6.4
22	Prime Led 3	8.1 ± 0.1	7.0 ± 0.1	449	343.4 ± 3.0	884.1 ± 7.7
23	Prime Led 3	8.1 ± 0.1	6.9 ± 0.1	446	327.6 ± 3.3	849.3 ± 8.6
24	Prime Led 3	7.9 ± 0.1	7.0 ± 0.1	447	371.6 ± 3.0	976.7 ± 7.8
25	Prime Led 3	7.9 ± 0.1	6.7 ± 0.1	448	331.9 ± 1.2	931.2 ± 3.4
26	Optilight Max	7.9 ± 0.1	6.9 ± 0.1	454	458.7 ± 4.0	1223.7 ± 10.7
27	Optilight Max	8.1 ± 0.1	6.2 ± 0.1	458	421.7 ± 9.6	1409.2 ± 31.9
28	Optilight Max	8.0 ± 0.1	6.7 ± 0.1	460	143.4 ± 1.2	403.3 ± 3.5
29	Optilight Max	7.9 ± 0.1	6.8 ± 0.1	451	186.0 ± 3.6	507.1 ± 9.8
30	LuxCler LED	7.7 ± 0.1	6.9 ± 0.1	442	204.8 ± 4.3	551.0 ± 11.7
31	DB686	7.8 ± 0.1	7.0 ± 0.1	457	235.1 ± 3.9	612.4 ± 10.1
32	DB686	8.1 ± 0.1	7.4 ± 0.1	458	137.2 ± 1.0	321.3 ± 2.4
33	DB686	8.1 ± 0.1	6.8 ± 0.1	451	94.2 ± 9.8	255.8 ± 26.6
34	Emitter A. fit	8.0 ± 0.1	6.8 ± 0.1	444	324.2 ± 5.9	889.0 ± 16.1
35	Emitter A. fit	8.1 ± 0.1	6.9 ± 0.1	446	391.6 ± 15.3	1058.0 ± 41.5
36	Emitter A. fit	8.1 ± 0.1	6.7 ± 0.1	450	431.8 ± 15.7	1230.4 ± 44.7
37	Emitter A. fit	8.0 ± 0.1	7.0 ± 0.1	446	448.0 ± 18.1	1180.0 ± 47.6
38	Emitter A. fit	8.1 ± 0.1	7.0 ± 0.1	445	367.4 ± 6.3	965.1 ± 16.6
39	Emitter A. fit	7.8 ± 0.1	6.1 ± 0.1	449	265.1 ± 9.7	911.3 ± 33.4
40	LD Max	8.3 ± 0.2	8.3 ± 0.2	443	147.2 ± 1.6	271.3 ± 2.9

### Radiant power and emission spectrum

**Figure 2:**
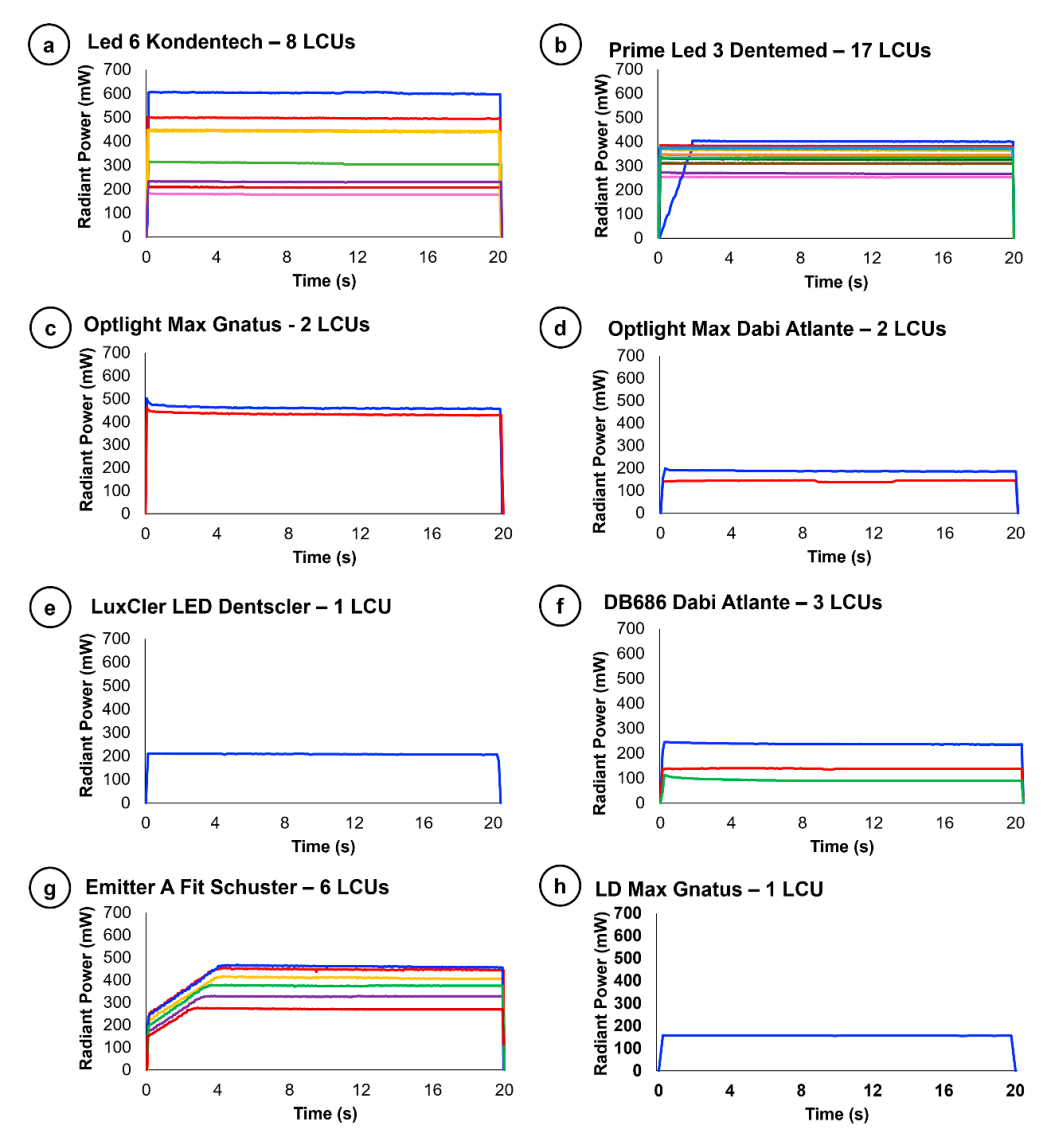
Radiant power (mW) emitted during a 20-second exposure from the 40 LCUs. A) LED 6; B) Prime Led 3; C) Optilight Max Gnatus; D) Optilight Max Dabi; E) LuxCler LED; F) DB686; G) Emitter A. fit; H) LD Max.

**Figure 3:**
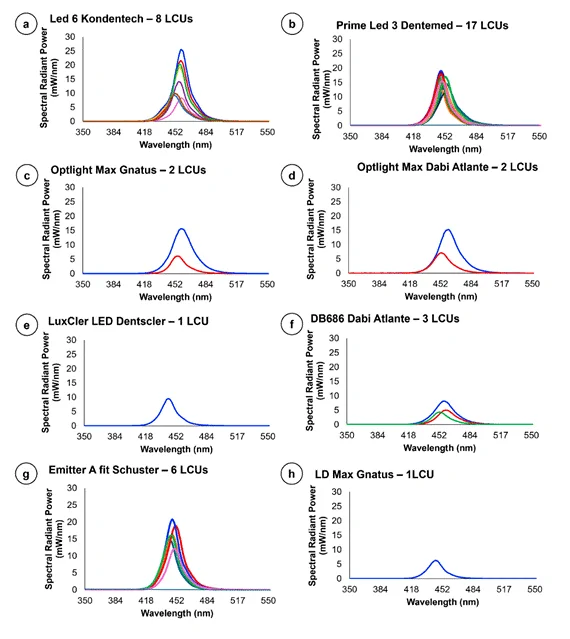
Spectral radiant power (mW/nm) from the 40 LCUs. A) LED 6; B) Prime Led 3; C) Optilight Max Gnatus; D) Optilight Max Dabi; E) LuxCler LED; F) DB686; G) Emitter A. fit; H) LD Max. Note the different spectra emitted from these 40 LCUs.

The radiant power ranged between 94.2 and 588.2 mW, and the calculated average irradiance of the LCUs ranged between 255.8 and 1597.8 mW/cm² (Table 3). Irradiance values were compared among the different LCU tip condition categories. No statistically significant differences were observed among groups (p = 0.632). However, LCUs with contaminated or damaged tips showed lower mean irradiance values than intact tips, suggesting a trend. Specifically, intact tips had the highest mean irradiance (937 mW/cm²), while contaminated (800 mW/cm²), damaged (904 mW/cm²), and both contaminated and damaged (864 mW/cm²) tips showed lower values. Considerable variability was observed within all groups. One-way ANOVA showed that radiant power values differed significantly across LCUs (P < 0.001). The radiant power (mW) profile showed large intra- and inter- variations among the LCUs. Most LCUs emitted stable radiant power; however, one Prime LED 3 had an affected emission on the initial 2 seconds (Figure 2). The Emitter A Fit ramped its radiant power output for the first 4 s, then stabilized (Figure 2). The peak wavelength emissions of the 40 LCUs are reported in Table 3. All the LCUs had a single-peak emission within the blue wavelength range (410-490 nm), but Figure 3 shows that the peak occurred at different wavelengths.

### Beam profile

**Figure 4:**
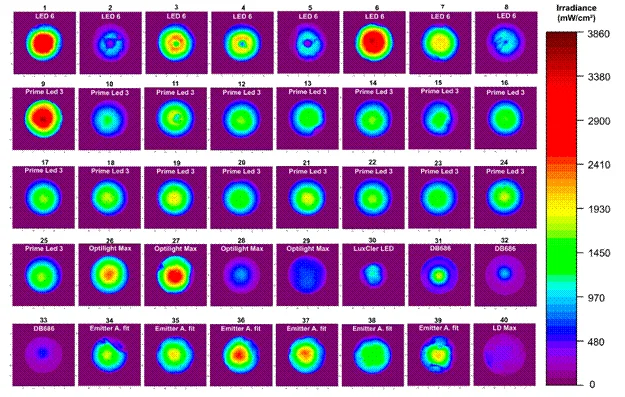
Two-dimensional light beam profiles from the LCUs show the irradiance (mW/cm²) at 0 mm distance without any interposing material. All the images were calibrated and displayed using the same spatial dimensions, and the irradiance scale was standardized across all images based on the highest recorded value.

Two-dimensional beam profiles for all LCUs at 0 mm distance are shown in Figure 4. Note the reduction in the light output area at the damaged tip edges and/or where the tip was contaminated with some residue. The light distribution is heterogeneous, and the irradiance is reduced over most of the LCU tip. LCUs code #1 (LED 6), #6 (LED 6), and #9 (Prime LED 3) emitted the highest peak irradiance, concentrated in the central region of the light tip. LCUs #3 (LED 6), #26 (Optilight Max), #27 (Optilight Max), #36 (Emitter A. fit), and #37 (Emitter A. fit) had peak irradiance concentrated in small central areas of the active tip. Most of the Prime LED 3 LCUs delivered a homogeneous light distribution; however, a lower emission was observed at the edges of their light tips.

## Discussion

The radiant power (94.2 mW to 588.2 mW) and irradiance (255.8 mW/cm² to 1597.8 mW/cm²) varied significantly among the LCUs used within the public health system; 85% showed damage (cracks, wear) and/or contamination by resinous residues (Figure 1). Therefore, the null hypotheses were rejected. These results revealed considerable variability among the LCUs and suboptimal maintenance practices. These findings may affect the clinical performance of the RBC restorations placed in the public health system in Brazil.

Variability in power and irradiance may be associated with different types of LCUs, duration of use, physical damage, contamination of tips, and lack of maintenance ^3^. In addition, as multiple LCUs were evaluated within the same health units, similarities in usage patterns and maintenance routines may have contributed to the observed variability, which should be considered when interpreting the results. Similar results have been reported in previous studies ^4^ ^,^ ^9^ ^,^ ^17^. Irradiance values below 500 mW/cm² have been associated with reduced polymerization effectiveness ^6^ ^,^ ^10^, while manufacturers often recommend minimum values around 1000 mW/cm² ^3^ ^,^ ^5^ ^,^ ^6^. However, it is important to recognize that adequate polymerization depends on multiple factors, including radiant exposure (energy), emission spectrum, beam uniformity, exposure time, and the optical properties and thickness of the restorative material. Although some recently introduced materials are claimed to require lower energy for polymerization, these claims depend on curing conditions and should be interpreted with caution. In this study, 5 LCUs emitted values below 500 mW/cm² (Table 3), which may negatively compromise polymerization and potentially increase the risk of premature failure of the restoration ^18^ ^,^ ^19^. Regular irradiance checks using radiometers are recommended ^20^ ^,^ ^21^ as a simple and effective protocol for clinicians and assistants. However, radiometers may vary in accuracy and spectral sensitivity and should therefore be used primarily to monitor changes in light output over time.

The two-dimensional beam profiles show that the light output was not uniform across the light tip (Figure 4), with higher irradiance in the central area of the tip and lower at the edges. This is particularly concerning in LCUs that deliver a low irradiance, compromising adhesion and marginal sealing, especially in larger restorations ^9^ ^,^ ^22^ ^,^ ^23^. LCUs with a small-diameter tip and beam concentrated at the center require multiple light exposures in different areas ^6^ for adequate photoactivation of the margins ^6^ ^,^ ^22^.

The physical examination of the light tips revealed a critical situation: 85% were damaged and/or contaminated (Figure 1), a condition known to reduce emitted power and irradiance ^11^ ^,^ ^13^ significantly. Although no statistically significant differences were observed among the LCU tip condition categories, LCU tips with contaminated or damaged tips tended to show lower irradiance values than intact LCU tips. This may be explained by the high variability among devices in this field-based evaluation. This high prevalence suggests deficiencies in adherence to LCU protection and hygiene protocols. All 16 health units evaluated across five regions of Uberlândia had LCUs with some problems. This finding can impact the effectiveness and longevity of RBC restorations ^6^ ^,^ ^23^, increase costs, and overload the system ^15^. Effective polymerization requires good LCU ^3^ ^,^ ^6^ ^,^ ^10^ ^),^ and model selection should consider power, irradiance, beam profile, curing time, ergonomics, and access to technical assistance ^21^. Cost-effectiveness analysis should consider not only the initial cost, but also durability, maintenance, and compatibility with RBCs ^3^ ^,^ ^8^ ^,^ ^21^. Although most LCUs were low-cost (US$ 101.80 and US$ 305.40), their regular use is indicated by an average of 132 monthly restorations per unit. This highlights the high clinical demand for these devices and reinforces the importance of ensuring adequate LCU performance. This investment represents a relatively low cost per restoration.

The emission spectra (Figure 3) show a single peak in the blue region (approximately 410-490 nm). The peaks were distinct across models and within the same model, such as the Led 6 (450-458 nm) and Prime Led 3 (446-452 nm). The peak range for all LCUs was consistent with that of camphorquinone (CQ), a widely used photoinitiator in dental RBCs ^8^. 

This study showed significant variability in brands, models, and technical characteristics, reflected in the acquisition model in the Brazilian public service ^24^: fragmented bidding processes, without standardization or technical planning. Standardizing LCUs is a strategic recommendation that simplifies maintenance, training, and monitoring their performance. Thus, based on the findings of this study and supported by literature, the following clinical recommendations for the acquisition, cleaning, maintenance, and use of LCUs are proposed:

### Technical criteria for the acquisition of new LCUs:

1. Irradiance ≥1000 mW/cm², to provide sufficient energy (16 to 20 J/cm²) and adequate polymerization of resin composites in a 20-second exposure ^3^ ^,^ ^6^.
2. Emission peak between 440-480 nm, the spectral range corresponding to the absorption peak of camphorquinone, the main photoinitiator present in light-curing composites ^23^.
3. A collimated and homogeneous beam to ensure uniform distribution of light output ^3^ ^,^ ^8^ ^,^ ^22^.
4. Avoid models with detachable optical fiber due to increased susceptibility to radiant power loss from optical misalignment or the accumulation of residue ^21^. This design is more susceptible to damage and tip breakage. When the tip is damaged, the LCU may become unusable or require costly, specialized repairs. This LCU model requires maintenance of both the LED and the light tip, increasing technical and logistical costs.
5. Integrated tip (fixed), LCUs with attached tips have greater robustness, resistance, and durability, especially in dental practices with high demand and continuous use ^21^.
6. Ergonomic design to optimize clinical handling and positioning of the LCU during procedures ^4^ ^,^ ^5^.
7. Digital timer with 10-40 second exposure timer options and auditory feedback. This enables more accurate control of exposure time during photoactivation.

### Protocol for cleaning and preventive maintenance:

1. Use a disposable plastic barrier, correctly applying it so there are no seams or folds over the light tip; plastic food film is a cost-effective alternative to protect the tip and body of the device ^12^.
2. Clean with 70% alcohol after each use, and store in a safe, dry place.
3. Periodic tip assessment (visual inspection), to check for physical damage, cracks, or broken optical fibers (which can show as black or dark areas on multifilament light tips). Check the LCU for contaminants after clinical use and remove them carefully. If the light tip, lens, or optical fiber is scratched or damaged, replace the component if possible, or return the unit for specialized repair ^11^ ^,^ ^13^.
4. Regular assessment of light output using a radiometer is recommended to detect reductions in radiant power. While most dental radiometers do not provide absolute measurements, they monitor changes in irradiance over time ^20^. Radiometers may vary in accuracy and spectral sensitivity and should therefore be used primarily to monitor changes in light output over time. If the light output has decreased by more than 20%, replacement of the unit should be considered, as reduced output may compromise radiant exposure ^3^ ^,^ ^6^. Reductions of up to 20% may be offset by increasing exposure time.
5. Keep a record of the light output and maintenance to simplify any follow-up or litigation.

### Clinical factors for use of LCUs:

1. Position the patient and yourself to improve your view of the work.
2. Watch what you are doing through protective orange glasses or a shield ^25^. If you look away, you cannot see what you are doing with the LCU.
3. Adjust the position of the light tip so that it remains perpendicular to the restoration surface and as close as possible to it ^4^.
4. When using a high-powered LCU, cool the tooth with a stream of air to prevent overheating ^4^ ^,^ ^6^.
5. Increase the light-curing exposure time to compensate for the reduction in light emitted by the LCU when a barrier is used ^4^.
6. When using LCU with a smaller tip diameter or a light beam concentrated at the tip center, perform multiple light exposures to cover the entire restoration fully.

The protocols proposed not only apply to public health but should be considered by all clinicians. Technical planning and equipment standardization are crucial for optimizing durability and clinical accuracy. LCUs used in public oral health units have significant variability and maintenance issues. The implementation of standardized protocols and the replacement of underperforming devices are important to ensure effective restorative treatments by the SUS. The limitations of this study are its observational design and its geographical delimitation to the city of Uberlândia. However, 16 public health units evaluated are a relevant number considering that Uberlândia has 45 public health units with dental services. Another limitation was that information on the duration and frequency of use (number of curing cycles) and the maintenance history of the LCUs was unavailable. These factors may have influenced device performance and partially explained the variability observed among the LCUs. However, this limitation guided the authors' orientation to the Health Administration of Uberlândia to implement the recording of this information. While extrapolation to other contexts is limited, these results corroborate the findings from previous studies ^13^ ^,^ ^14^. The performance of LCUs used in all dental practices must be continually examined. Multicenter studies are needed to understand the performance of LCUs and their impact on the quality of restorations. These findings reinforce the importance of routine monitoring and maintenance of LCUs in clinical practice.

## Conclusions

Within the limitations of this observational field study, it is possible to conclude that 85% of the LCUs evaluated had damaged or contaminated light tips, potentially compromising the quality and uniformity of the emitted light. There was significant variation in radiant power and irradiance among the different LCU models, and the light beam distribution was heterogeneous, with a concentration of irradiance in central areas and a reduction at the edges, particularly at damaged or contaminated tips. 

## Data Availability

The research data are available upon request.
